# Distinct effects of SIRT1 in cancer and stromal cells on tumor promotion

**DOI:** 10.18632/oncotarget.8073

**Published:** 2016-03-14

**Authors:** Dong Hoon Shin, Yong-Joon Choi, Peng Jin, Haejin Yoon, Yang-Sook Chun, Hyun-Woo Shin, Ja-Eun Kim, Jong-Wan Park

**Affiliations:** ^1^ Department of Pharmacology, Seoul National University College of Medicine, Seoul 110-799, Republic of Korea; ^2^ Lung Cancer Branch, Division of Translational & Clinical Research, Department of System Cancer Science, Graduate School of Cancer Science and Policy, National Cancer Center, Gyeonggi-do 410-769, Republic of Korea; ^3^ Department of Biomedical Sciences, Seoul National University College of Medicine, Seoul 110-799, Republic of Korea; ^4^ Ischemic/Hypoxic Disease Institute and Cancer Research Institute, Seoul National University College of Medicine, Seoul 110-799, Republic of Korea; ^5^ Department of Pharmacology, School of Medicine, Kyung Hee University, Seoul 02447, Republic of Korea

**Keywords:** SIRT1, tumor, stroma, MMP3

## Abstract

The lysyl deacetylase SIRT1 acts as a metabolic sensor in adjusting metabolic imbalance. To explore the role of SIRT1 in tumor-stroma interplay, we designed an *in vivo* tumor model using SIRT1-transgenic mice. B16F10 mouse melanoma grew more quickly in SIRT1-transgenic mice than in wild-type mice, whereas SIRT1-overexpressing one grew slowly in both mice. Of human tumors, SIRT1 expression in stromal fibroblasts was found to correlate with poor prognosis in ovarian cancer. B16F10 and human ovarian cancer (SKOV3 and SNU840) cells were more proliferative in co-culture with SIRT1-overexpressiong fibroblasts. In contrast, SIRT1 within cancer cells has a negative effect on cell proliferation. In conditioned media from SIRT1-overexpressing fibroblasts, matrix metalloproteinase-3 (MMP3) was identified in cytokine arrays to be secreted from fibroblasts SIRT1-dependently. Fibroblast-derived MMP3 stimulated cancer cell proliferation, and such a role of MMP3 was also demonstrated in cancer/fibroblast co-grafts. In conclusion, SIRT1 plays differential roles in cancer and stromal cells. SIRT1 in stromal cells promotes cancer growth by producing MMP3, whereas SIRT1 in cancer cells inhibits growth via an intracellular event. The present study provides a basis for setting new anticancer strategies targeting SIRT1.

## INTRODUCTION

Cancer cells live and act in conjunction with stromal cells residing in the vicinity of tumors or immigrating from bone marrow [1, 2]. Cancer-associated fibroblasts (CAFs), which are the most populous stromal cells, are not innocent neighbors but rather active assistants for cancer [3, 4]. Cancer cells and CAFs secrete many cytokines that stimulate the cooperative growth and activation among them [5]. Therefore, cancer-stroma interplay should be examined to better understand tumor biology.

Sirtuins belong to the class III histone deacetylases that utilize NAD^+^ to remove the acetyl moiety from lysine. Of them, SIRT1 has been most intensively investigated in terms of its biological functions [6]. SIRT1determines gene expression by deacetylating either histones or transcription factors [7, 8]. SIRT1 participates in a variety of biological processes, such as metabolic reprogramming, cell proliferation, differentiation, and senescence [9, 10]. However, the role of SIRT1 in cancer has not been clearly understood. SIRT1 suppresses tumor formation and growth by preventing genotoxic stress and inducing apoptosis [11, 12]. Conversely, it promotes tumor growth by inhibiting p53 and Foxo3a, and also facilitates tumor expansion by inducing epithelial-to-mesenchymal transition [13–15].

Our main concern is why there are conflicting reports regarding the role of SIRT1 in cancer. We suggest that this confusion arises from the fact that SIRT1 has not been explored based on the concept of tumor-stroma interplay. Therefore, we here designed an *in vivo* tumor model using SIRT1-transgenic mice and a co-culture model of cancer and stromal cells. We concluded that cancer growth is promoted by SIRT1 in stromal cells but demoted by SIRT1 in cancer cells.

## RESULTS

### Differential roles of cancer SIRT1 and host SIRT1 in growth of tumor grafts

**Figure 1:**
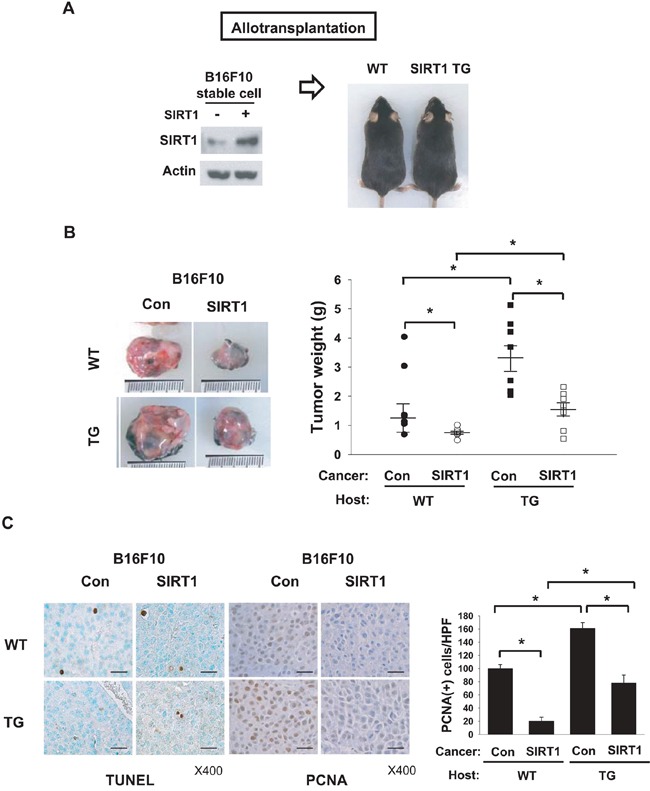
Effects of SIRT1 expressed in cancer and host cells on tumor growth **A.** Scheme for allotransplantation of B16F10 stable cell lines into wild type (WT) and SIRT1-transgenic (TG) mice. Left and right panels show immunoblotting of SIRT1 in B16F10 cell lines and photographs of WT and SIRT1-TG littermates, respectively. **B.** Control and SIRT1-expressing B16F10 stable cell lines were implanted into the flanks of WT and TG mice. Tumors were excised (left) and weighed (right) on day 14 after transplantation. All results are shown as the mean ± s.e.m. (n=8; right). *, p < 0.05. **C.** TUNEL analysis and PCNA staining were performed in tumor tissue sections (left). Numbers of PCNA-positive cells per high-power field (HPF) at 400× magnification (means + s.d.; n=4) are shown (right). *, p < 0.05; scale bar, 100 μm.

Control and SIRT1-overexpressing B16F10 stable cell lines were grafted into wild type (WT) or SIRT1-transgenic (TG) mice (Figure 1A). All grafts successfully established growing tumors in mice, suggesting that SIRT1 overexpression does not negate the tumorigenic potential of cells. SIRT1-overexpressiong tumors grew more slowly than control tumors in both WT and TG mice (Figure 1B). Comparing tumor growth between WT and TG mice, control and SIRT1-overexpressing tumors both grew more quickly in TG mice (Figure 1B). As a tumor achieves a proliferative state by acquiring self-sufficiency in cell growth and/or by becoming resistant to cell death [16], we analyzed the expression of PCNA (proliferation index) and TUNEL (death index). PCNA expression correlated with tumor weight in all groups, whereas TUNEL positivity was not different among groups (Figure 1C). These results suggest that SIRT1 in host stromal cells provides a tumor-favorable environment, whereas SIRT1 in cancer cells has a negative effect on tumor growth.

### Correlation between stromal SIRT1 expression and poor outcome of ovarian cancer patients

**Figure 2:**
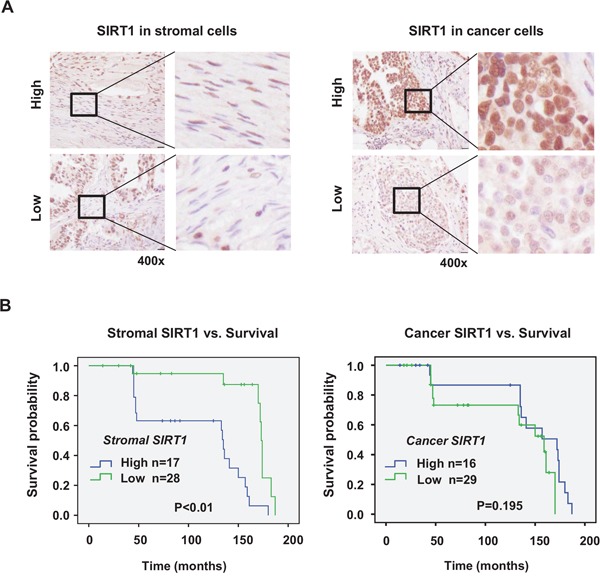
Survival analysis of ovarian cancer patients SIRT1 expression was immunohistochemically analyzed in cancer cells and stromal cells of each specimen in human ovarian cancer tissue microarray. Representative images (400x magnification) of immunostained SIRT1 in stromal fibroblasts and cancer cells of human ovarian cancer tissues are presented in the panel **A.** The scale bar represents 20 μm. Quantification of protein expression by estimation of staining intensity was described in Materials and Methods. **B.** Kaplan–Meier curves were plotted to estimate overall survival of patients with cancer according to the expression of SIRT1. Significant association of SIRT1 expression with overall survival was analyzed by comparing differences between curves using the log-rank test.

To examine the role of SIRT1 in human cancer progression, we checked the relationship between SIRT1 expression and survival of patients with ovarian cancer. Ovarian cancer tissues were chosen as appropriate specimens for this experiment because ovarian cancer had a high mortality rate and contains fibrotic areas demarcated clearly in histology. SIRT1 expression was evaluated separately in stromal fibroblasts and cancer cells (Figure 2A). The high expression of SIRT1 in fibroblasts is associated with poor prognosis in this cancer population (Figure 2B). Although the SIRT1 expression in cancer cells tends to correlate with patients' survival, two survival curves are not statistically different. The results support our notion that SIRT1 in stromal cells promotes cancer progression, but the clinical consequence of SIRT1 expression in cancer cells remains to be further investigated.

### Fibroblasts stimulate cancer growth SIRT1-dependently

**Figure 3:**
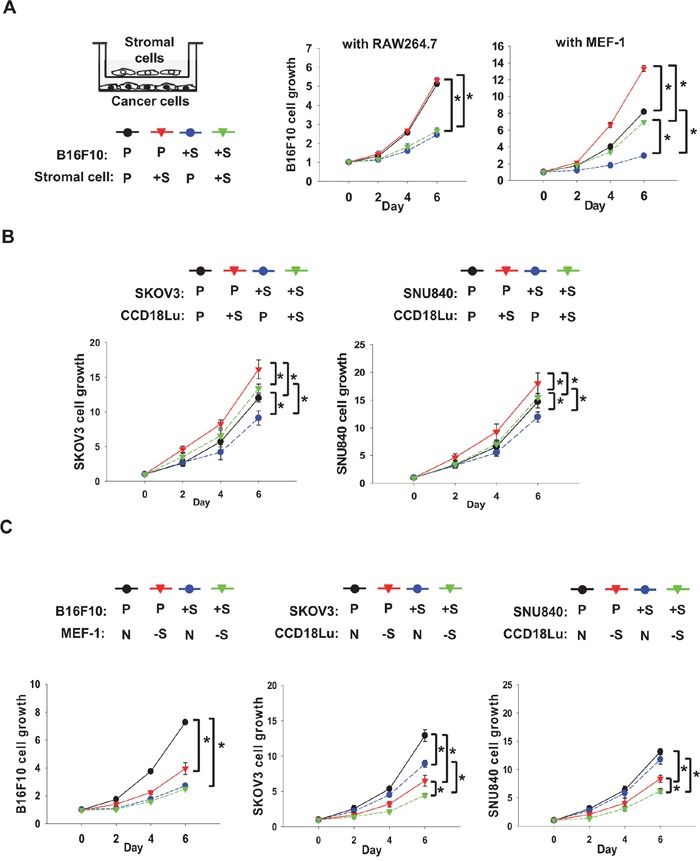
Role of SIRT1 in proliferation of cancer cells co-cultured with fibroblasts **A.** RAW264.7, MEF-1, and B16F10 cells, which had been transfected with the indicated plasmid (2 μg/100 mm dish), were co-cultured in Boyden chambers (stromal cells in the upper chamber and cancer cells in the lower chamber). **B.** Human ovarian cancer cells (SKOV3 and SNU840) and human fibroblasts (CCD18Lu), which had been transfected with the indicated plasmids, were co-cultured in Boyden chambers. **C.** B16F10, SKOV3, SNU840, MEF-1, and CCD18Lu cells, which had been transfected with the indicated siRNAs (80 nM), were co-cultured in Boyden chambers. Cell growth was analyzed using MTT. Data (mean ± s.d.; n=4) are plotted as a function of incubation time. *, p < 0.05. P, +S, N, and -S denote the transfection with pcDNA, SIRT1 plasmid, non-targeting RNA, and SIRT1-targeting siRNA, respectively.

To examine the different roles of SIRT1 in cancer and stromal cells, B16F10 cancer cells and one of two types of stromal cells were co-cultured in trans-well chambers. Since macrophages and fibroblasts are regarded as major stromal cells affecting tumor growth or invasion [17, 18], RAW264.7 mouse macrophage and MEF-1 mouse fibroblast cell-lines were used as stromal cells. SIRT1 overexpression or knock-down in these cell-lines was verified by immunoblotting (Supplementary Figure 2). When B16F10 and RAW264.7 were co-cultured, B16F10 proliferation was not affected by SIRT1 overexpression in RAW264.7, but was significantly inhibited by that in B16F10. In co-culture conditions, however, B16F10 proliferation was substantially increased by SIRT1-overexpressing MEF-1 and, as expected, was retarded by SIRT1 overexpression in B16F10 (Figure 3A). We next co-cultured human cell lines of fibroblast (CCD18Lu) and ovarian cancer (SKOV3 and SNU840). The growth rates of both ovarian cancer cells were raised by co-culture of SIRT1-overexpressing CCD18Lu cells, whereas they were decreased by intracellularly overexpressed SIRT (Figure 3B). In addition, the growth of these cancer cells was inhibited in co-culture with MEF-1 or CCD18Lu fibroblasts where SIRT1 was knocked down (Figure 3C). SIRT1-overexpressing cancer cells exhibited slow growth, and the growth was further inhibited in co-culture with SIRT1 knocked-down fibroblasts (Figure 3C).

### SIRT1-overexpressing fibroblasts release a paracrine factor stimulating cancer growth

**Figure 4:**
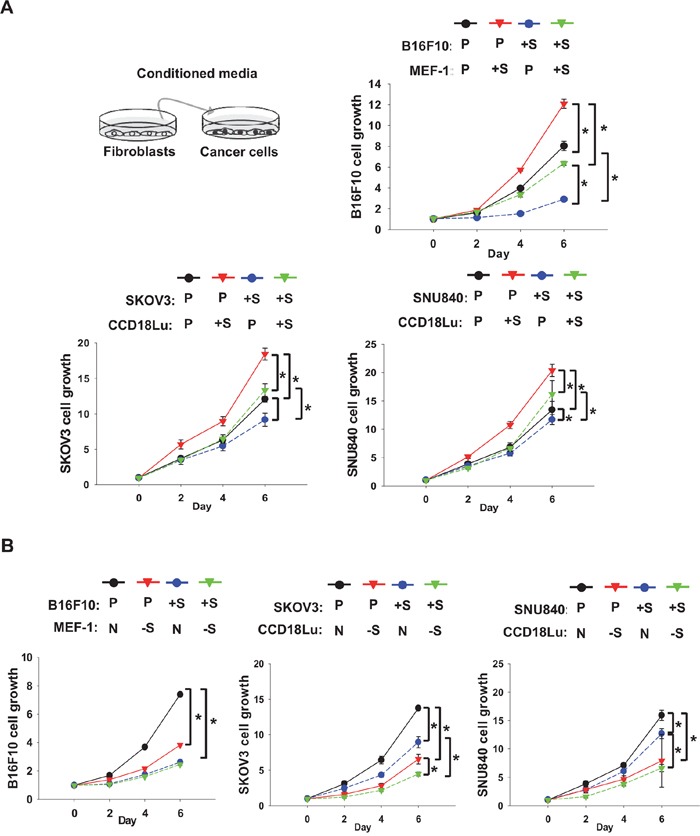
Role of fibroblast SIRT1 in cancer cell proliferation Cancer cells (B16F10, SKOV3, and SNU840) and fibroblasts (MEF-1 and CCD18Lu) were transfected with plasmids **A.** or siRNAs **B.** as described in Figure 3. Cancer cells were cultured in a 1:1 (v/v) mixture of conditioned medium from fibroblasts and fresh medium. Cell growth was analyzed using MTT. Data (mean ± s.d.; n=4) are plotted as a function of incubation time. *, p < 0.05. P, +S, N, and -S denote the transfection with pcDNA, SIRT1 plasmid, non-targeting RNA, and SIRT1-targeting siRNA, respectively.

**Figure 5:**
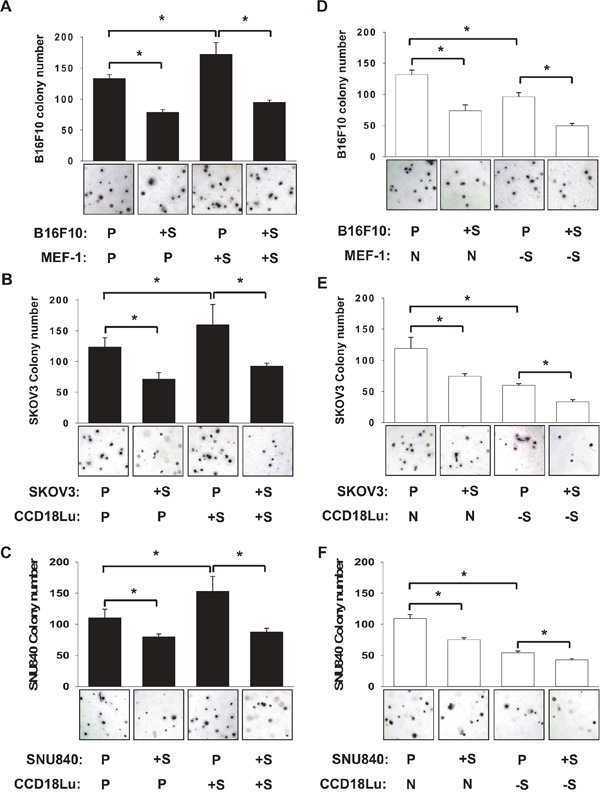
Role of fibroblast SIRT1 in cancer cell colonization B16F10 **A, D.** SKOV3 **B, E.** or SNU840 **C, F.** cancer cells, which had been transfected with pcDNA or SIRT1 plasmid, were seeded on 0.4% top agar and cultured in the mixture (1:1) of fresh medium and conditioned medium from MEF-1 or CCD18Lu fibroblasts which had been transected with plasmids (empty vector or SIRT1) or siRNAs (non-targeting or SIRT1-targeting). On day 21 after cell seeding, cell colonies were visualized using crystal violet and counted. Data (number of colonies per 3.7 cm^2^) are presented as the mean + s.d. (n=4). *, p < 0.05. P, +S, N, and -S denote the transfection with pcDNA, SIRT1 plasmid, non-targeting RNA, and SIRT1-targeting siRNA, respectively.

To examine whether fibroblasts promote cancer proliferation via paracrine factors, we applied the media from fibroblast culture to cancer cells. The growth of B16F10, SKVO3, or SNU840 cells was increased in conditioned media from SIRT1-overexpressing MEF-1 or CCD18Lu cells, and the growth of SIRT1-overexpressing cancer cells was further retarded in the media (Figure 4A). When these cancer cells were incubated in the media from SIRT1-deficeint fibroblasts, the growth rate was significantly declined (Figure 4B). In a colony-forming assay reflecting anchorage-independent cancer growth, cell colonization within agar matrix was inhibited in cancer cells overexpressing SIRT1. However, the colonization was facilitated in the conditioned media from SIRT1-overexpressing fibroblasts, which was commonly shown in three different cancer cell lines (Figure 5A-5C). Conversely, when cancer cells were cultured in the conditioned media from SIRT1-deficient fibroblasts, the number of cancer colonies was significantly diminished (Figure 5D-5F). Fibroblasts may release some cancer-proliferating factor(s) SIRT1-dependently, but SIRT1 may provoke an intracellular event to inhibit cancer growth. Growth inhibition by intracellular SIRT1 was also shown in MEF-1 and CCD18Lu fibroblasts (Supplementary Figure 3).

Next, we investigated whether the SIRT1-dependent paracrine factor from fibroblasts affects proliferation in other cancer cell-lines. The growth of RCC4 was greatly increased in the medium conditioned by SIRT1-overxpressing MEF-1, whereas the growth of other cell-lines was marginally or not stimulated in this medium (Supplementary Figure 4). The medium conditioned by SIRT1-overexpressing NIH3T3 fibroblasts also increased RCC4 proliferation, and did so to a lesser extent in other cell-lines (Supplementary Figure 5). Collectively, these results suggest that some SIRT1-dependent, fibroblast-derived factors stimulate cancer proliferation depending on the cell context.

### MMP3 is an SIRT1-dependent, fibroblast-derived factor stimulating cancer proliferation

**Figure 6:**
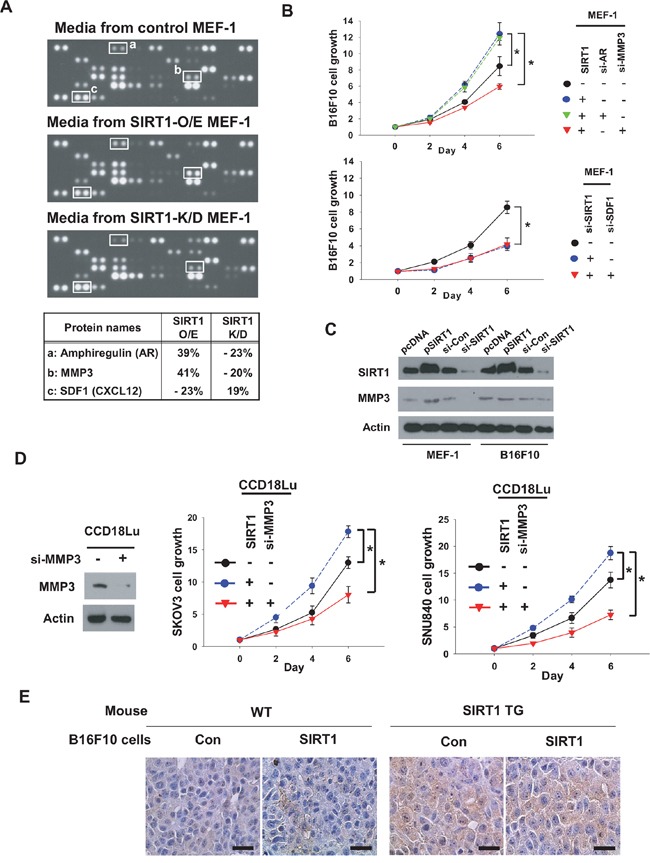
MMP3 is SIRT1-dependently secreted from fibroblasts and stimulates cancer cell growth **A.** Conditioned media were collected from MEF-1 cells transfected with SIRT1 (2 μg) or siSIRT1 (80 nM). The media were applied to ARY015 mouse antibody arrays. Representative array images are shown in the upper three panels. Three SIRT1-dependently secreted factors are listed in the bottom panel. **B.** MEFs and B16F10 cells were transfected as indicated and then co-cultured. **C.** MEF-1 and B16F10 cells were transfected with 2 μg of empty vector or SIRT1 plasmid, or 80 nM siRNA targeting none or SIRT1. After incubated for 48 h, cells were lysed to immunoblot the indicated proteins. **D.** SKOV3, SNU840, and CCD18Lu cells were transfected and co-cultured as indicated. Cell growth (mean ± s.d.; n=4) was analyzed by MTT staining. *, p < 0.05. E, MMP3 levels in B16F10 tumors (see Figure 1) were analyzed by immunohistochemistry. Representative images were captured under a microscope at 400× magnification (scale bar, 100 μm).

**Figure 7:**
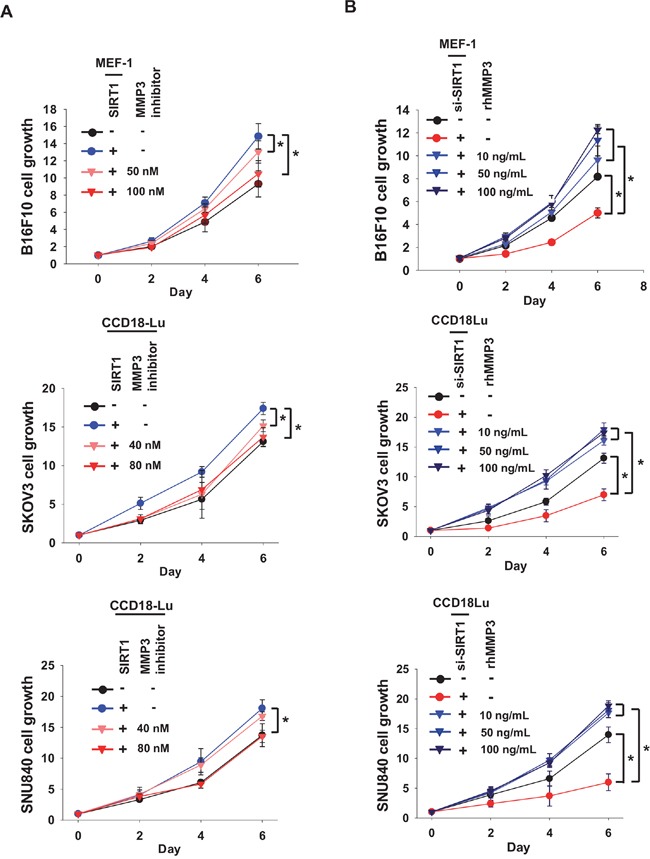
MMP3 acts as a growth factor for cancer cells **A.** Cancer cells (B16F10, SKOV3, or SNU840) cells were co-cultured with fibroblasts (MEF-1 or CCD18Lu) which were transfected with SIRT1. MMP3 inhibitor at the indicated concentrations was administered into the co-culture media. **B.** Conditioned media were collected from fibroblasts transfected with si-SIRT1 (80 nM). Cancer cells were cultured in a 1:1 (v/v) mixture of the conditioned medium and fresh medium, and treated with recombinant human MMP3 (rhMMP3) at the indicated concentrations. Cancer cell growth was estimated using MTT. Data (mean ± s.d.; n=4-6) are plotted as a function of incubation time. *, p < 0.05.

To search for fibroblast-derived factors responsible for cancer proliferation, we applied conditioned media from control, SIRT1-overexpressing, and SIRT1-deficient MEF-1 cells to protein profiling arrays (Figure 6A), and compared the intensities of spots among three groups. Subsequently, we chose three candidates whose levels depended on SIRT1 expression (Supplementary Figure 6A), namely, amphiregulin (AR), matrix metalloproteinase-3 (MMP3) and stromal cell-derived factor-1 (SDF1). The SIRT1-dependent expression of these factors was confirmed by immunoblotting (Supplementary Figure 6B, lanes 1-2 in each panel). Based on the knock-down efficiencies of siRNAs (three for each mRNA), we selected si-AR_1, si-MMP3_3, and si-SDF1_1 for the following experiments (Supplementary Figure 6B, lanes 3-5 in each panel). To examine whether SIRT1-induced AR and MMP3 promote cancer proliferation, we co-cultured B16F10 with MEF-1 treated with siRNAs targeting them. Interestingly, the cancer proliferation effect of MEF-1-conditioned medium was abolished by MMP3 knock-down, but not by AR knock-down (Figure 6B, top). Because SDF1 is induced by SIRT1 knock-down, we tested the possibility that SDF1 is responsible for B16F10 growth retardation by SIRT1 knock-down in MEF-1. The effect of SIRT1 knock-down was not reversed by SDF1 inhibition (Figure 6B, bottom). Of three candidates, MMP3 alone was identified to increase cancer proliferation. The SIRT1-dependent expression of MMP3 was also confirmed by checking MMP3 levels in MEF-1 cells that overexpress or lack SIRT1 (Figure 6C). In B16F10 cancer cells, however, the MMP3 level was found to be consistent regardless of SIRT1 expression. This suggests that the role of SIRT1 in MMP3 expression is variable depending on cell context. The positive role of CCD18Lu-derived MMP3 in cancer cell proliferation was also verified in SKOV3 and SNU840 cell-lines (Figure 6D). Indeed, MMP3 was shown to be expressed in grafted tumors, depending on host SIRT1 expression (Figure 6E). To address the possibility that MMP3 is a new target for blocking cancer-fibroblast interplay, the MMP-3 inhibitor was administered into co-culture media at concentrations (< 100 nM for mouse cell-lines, < 80 nM for human cell-lines) that do not affect the growth of fibroblasts and cancer cells for 6 days (Supplementary Figure 7). The growth of three cancer cell-lines in co-culture with fibroblasts was retarded by MMP3 inhibitor (Figure 7A). Reversely, the cell growth, which was retarded by co-culture with SIRT1 knock-down fibroblasts, was recovered by a recombinant MMP3 peptide (Figure 7B). Given that some of MMP enzymes are known to promote the invasion of cancer cells, we examined whether MMP3 also has such the effect on B16F10 cells. As expected, the recombinant peptide of MMP3 was shown to robustly stimulate the invasion of B16F10 in a trans-well system (Supplementary Figure 8).

### MMP3 mediates tumor promotion by fibroblast SIRT1

**Figure 8:**
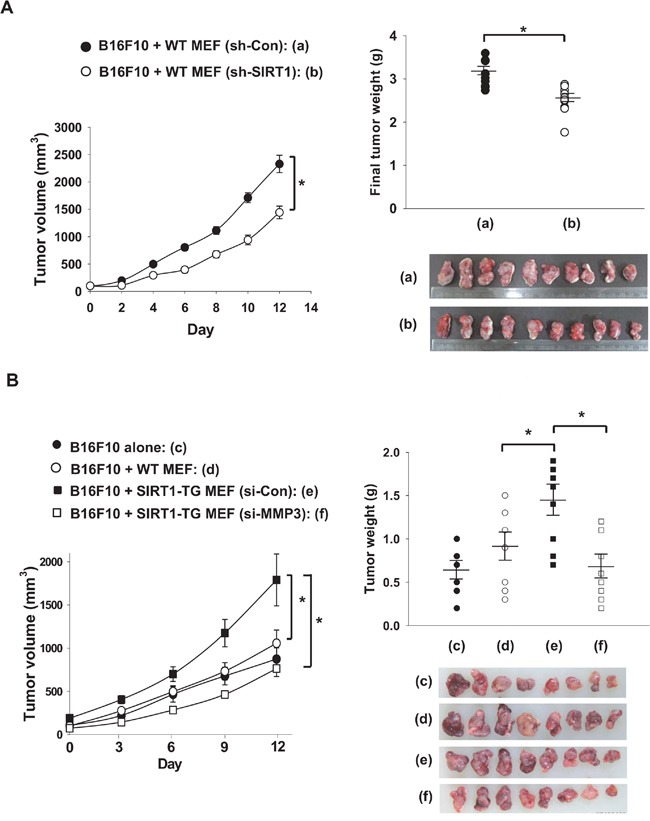
Fibroblast-derived MMP3 promotes tumor growth **A.** MEF cells obtained from WT mice were infected with sh-LTviral-Control or -SIRT1 (0.9 × 108 TU/mL) for 72 hour. The MEF cells (1×10^5^) and B16F10 cells (1×10^6^) were mixed with Matrigel and injected into the flanks of nude mice. Tumor volumes are expressed at the means ± s.e.m. (n, 10; *, p < 0.05) in the left panel. Tumors (a, b) were excised and weighed on the final day. Each tumor weight is plotted in the right panel and pictures of tumors are shown below the plot. **B.** MEFs obtained from WT and SIRT1-TG mice were transfected with the indicated siRNAs (80 nM). MEFs (1×10^5^) and B16F10 cells (1×10^6^) were mixed with Matrigel and the cell mixtures were injected into the flanks of nude mice. Tumor volumes were measured from day 7 after implantation. Results are expressed as the means ± s.e.m. (n, 6-8; *, p < 0.05) in the left panel. Each tumor weight is plotted in the right panel and pictures of tumors are shown below the plot.

To examine the role of fibroblast SIRT1 in tumor growth, B16F10 cells were co-grafted with SIRT1 knock-down MEFs, and the tumor growth was retarded (Figure 8A, left). B16F10 tumors excised from mice were weighed (Figure 8A, right), which verifies the tumor growth retardation in co-graft with SIRT1 knock-down MEFs. The SIRT1 downregulation by shRNA was checked using Western blotting (Supplementary Figure 9), which also shows the SIRT1-dependent secretion of MMP3. To evaluate the possibility of MMP3-targeted anticancer therapy *in vivo*, B16F10 cells were co-grafted into mice with MEFs that were isolated from WT or TG mice. It has been reported that in co-graft models, a large number of fibroblasts can promote tumor growth by providing a tumor-favorable microenvironment [19]. If so, the SIRT1-specific effect on tumor growth could be masked by this bystander effect. Therefore, we injected B16F10 with a small number of MEFs (10% of B16F10 number). We observed no effect of WT MEF co-grafts on tumor growth (Figure 8B). However, growth of B16F10 tumors was faster with SIRT1-TG MEFs, and was significantly attenuated by MMP3 knock-down (Figure 8B, left). We also weighed all B16F10 tumors excised from mice (Figure 8B, right). These results suggest that fibroblast-derived MMP3 is a potential target for cancer therapy.

## DISCUSSION

The controversy over whether SIRT1 promotes or inhibits tumor promotion has not been resolved so far. In the present study, we first explored the roles of SIRT1 in tumor growth based on the concept of cancer-stroma interplay. Briefly, fibroblast SIRT1 promotes tumor growth by enforcing cancer-stroma interplay, whereas cancer SIRT1 inhibits tumor growth. Furthermore, MMP3 is SIRT1-dependently secreted from fibroblasts and facilitates tumor growth. Our study provides a better understanding of the controversial roles of SIRT1 in cancer promotion and also an important consideration in developing SIRT1-targeting anticancer agents.

SIRT1 activates or inactivates multiple transcription factors [20]. Considering the diverse targets of SIRT1, it is not surprising that SIRT1 can act as a tumor promoter or suppressor depending on cell context. In our experimental settings, SIRT1 in cancer cells is characterized to play a tumor suppressive role, but the precise action of SIRT1 in cancer was not further investigated. According to previous literatures, it is speculated that SIRT1 inhibits cell growth by targeting the Wnt/β-catenin, NF-κ B, or HIF-1 signaling pathway [21–23]. As β-catenin is constitutively activated in many human tumors including ovarian cancer and melanoma [24], SIRT1 may inhibit the growth of these tumors by deacetylating and inactivating β-catenin [21]. In addition, SIRT1 deacetylates and inactivates NF-κB [22], which plays a beneficial role in cancer survival under stressful conditions like inflammation, and HIF-1α [23], which promotes cancer survival under hypoxic microenvironment. Accordingly, the inhibition of some of these signaling pathways may be associated with the inhibitory action of SIRT1 on cancer growth.

MMPs are regarded as important players mediating the cancer-stroma interaction [25]. Because MMPs loosen extracellular matrix, they promote tumor angiogenesis, invasion and metastasis. Beyond such actions, they can provoke cancer growth-promoting signals. For example, MMPs increase the interstitial levels of growth factors by converting precursors to active forms and by releasing them from tight matrix [26]. MMPs also stimulate integrin-mediated cell proliferation [27]. Notably, MMP3, alternatively named stromelysin-1, is regarded as a stromal MMP that exerts tumor-promoting effects in mammary, colorectal, and ovarian cancers [28–30]. Such an effect of MMP3 was also demonstrated in this study. MMP3 might be a potential anticancer target to block the tumor-stroma interplay.

The production of MMP3 is stimulated in fibroblasts by SIRT1. Based on the sequence of the MMP3 promoter, the transcription of the MMP3 gene may be determined by various transcription factors, such as Ets, TCF/β-catenin, STAT, AP1, and HIF-2 [31]. Of them, β-catenin and AP1 are known to be inactivated by SIRT1 [21, 32], but HIF-2 to be activated by SIRT1 [33]. In addition, the MMP3 expression is repressed epigenetically through the CpG methylation of its promoter or the chromatin remodeling [34]. Therefore, the way that SIRT1 regulates the MMP3 promoter cannot be easily expected. Indeed, Ohguchi *et al*. demonstrated that SIRT1 inhibits the interleukin-1β-induced expression of MMP1 and MMP3 at the transcriptional level in skin fibloblasts [35], which was a contradictory finding to ours. Although we cannot explain this discrepancy clearly, the regulation of MMP3 expression may be variable depending on cell context.

## MATERIALS AND METHODS

### Materials

The plasmid of Myc/His-tagged Sirt1 was constructed as described previously [23]. SIRT1-targeting lentivirus (SH3001-07) and non-targeting lentivirus were purchased from ATCGbio Life technology Inc. (Vancouver, Canada). The nucleotide sequences of siRNAs used are summarized in Supplementary Table 1. MMP-3 inhibitor (sc-311431) and antibodies against SIRT1, Myc tag, Amphiregulin, MMP3, SDF1, and β-actin were obtained from Santa Cruz Biotechnology (Santa Cruz, CA). A recombinant peptide of human MMP-3 (513-MP-010) was purchased from R&D Systems (Minneapolis, MN).

### Cell culture

B16F10 (mouse melanoma), MEF-1 (mouse embryonic fibroblast), NIH3T3 (mouse embryonic fibroblast), CCD18Lu (human lung fibroblast), RCC4 (human renal cancer), HCT116 (human colon cancer), U-87MG (human glioblastoma), and A549 (human lung cancer) cell lines were obtained from the American Type Culture Collection (Manassas, VA), and SKOV3 (human ovarian cancer) and SNU840 (human ovarian cancer) from the Korean Cell Line Bank (Seoul, Korea). The cells were cultured in MEM, DMEM, or RPMI1640, supplemented with 10% heat-inactivated fetal bovine serum, at 37°C in a 5% CO_2_ chamber. To obtain primary MEFs, mouse E13.5 embryos were removed and dissected to remove the heads and internal organs. The embryo trunks were digested with 0.25% trypsin in a 5% CO_2_ chamber at 37°C for 5 min. Cells were dispersed, centrifuged, and resuspended in DMEM. Co-culture was performed in 24-Transwell plates (Corning Costar, Cambridge, MA). Cancer cells and fibroblasts were seeded at the same density (6×10^3^/well) in the lower and upper chambers, respectively. Conditioned medium was collected from 3 day-cultured MEF-1 cells, and mixed with the same volume of fresh medium, which was transferred to cancer cells.

### Transfection and establishment of stable cell lines

For transient overexpression or knock-down, B16F10 or MEF-1 cells at 40% density were transfected with plasmids (2 μg per 100-mm dish) or siRNAs (80 nM) using Lipofectamine^®^ 2000 (Life Technologies). The transfected cells were allowed to be stabilized for 48 hours before experiments. To establish stable transfectant cell lines, B16F10 cells at 40% density were transfected with 2 μg of plasmid using Lipofectamine^®^ 2000. After stabilized for 36 hours, cells were cultured in the presence of 0.45 mg/ml of G418. Fifteen colonies from three different transfections were pooled to avoid gene expression bias due to variable chromosomal integration.

### Generation of SIRT1-transgenic mice

Transgenic mice were created by injecting a CMV promoter-SIRT1-Myc/His vector (Supplementary Figure 1A) into fertilized eggs from C57BL6 mice. Of 16 transgenic lines, line #4 was used in this study. Genomic DNA from tails was subjected to genotyping (Supplementary Figure 1B). SIRT1 levels in major organs were checked by immunoblotting with anti-Sirt1 and anti-6xHis (Supplementary Figure 1C). Because the immunological status can affect tumor graft, leukocytes were counted, indicating that the leukocyte profile is not altered by transgenic SIRT1 (Supplementary Figure 1D). All experiments were approved by the Institutional Animal Care and Use Committee (approve #SNU-120313).

### Tumor transplantation in mice

SIRT1-transgenic mice or nude mice (BALB/cAnNCrj-nu/nu), purchased from Charles River (Shin-Yokohama, Japan), were injected at a dorsal flank site with 1×10^6^ cancer cells and/or 1×10^5^ MEFs suspended in PBS or Matrigel. Tumor volume (length×width^2^×0.52) was measured with calipers for 14 days. Tumors were cut into two, and fixed or frozen. All animal procedures were performed in accordance with a protocol approved by the Institutional Animal Care and Use Committee (approve #SNU-130104).

### Cell growth and colony-forming assays

Cells (2×10^3^/well) were plated in 96-well plates and incubated with the MEF-conditioned medium. Cells were stained with MTT dye to measure viability. Quadruplicate wells were used for each analysis and data were obtained from at least three independent analyses. To analyze the anchorage-independent cancer growth, cells (2×10^3^/well) were suspended in 0.4% top agar and cultured on 0.8% agar for 21 days. Cells were stained with crystal violet, and cell masses (>0.2-mm diameter) were counted as colonies.

### Protein profiling arrays

Culture supernatants were applied to ARY015 mouse antibody arrays (R&D Systems) overnight at 4°C. The arrays were incubated overnight with a biotinylated antibody cocktail, and further incubated with HRP-conjugated streptavidin. The membrane was treated with a chemiluminescent detection reagent and the signals were visualized on an X-ray film. Spot intensities were analyzed using ImageJ software (NIH, Bethesda, MD).

### Cancer tissue array and immunohistochemistry

We purchased human ovarian cancer tissue arrays from SuperBioChips (Seoul, Korea). The tissue array contained 45 ovarian cancer tissues and also provided information on survival and cause of death. The specimens were deparaffinized, autoclaved to retrieve antigens, and sequentially incubated with 3% H_2_O_2_, anti-SIRT1 antibody (1:100; Santa Cruz Biotechnology) and a biotinylated secondary antibody (1:200; Vector Laboratories). The immune complexes were visualized using Vectastatin ABC (Vector), and the sections were counterstained with hematoxylin. The expression level was scored as four grades based on percentage of immunopositive cells: +/− (<20%), + (20~49%), ++ (50~80%), and +++ (>80%). The low group included +/− and +, and the high group did ++ and +++.

### Statistical analysis

Each result is expressed as the mean and standard deviation (s.d.) or standard error of the mean (s.e.m.), which were calculated using Microsoft Excel 2010. Groups were compared using two-tailed, unpaired Student's *t* test and p<0.05 was considered significant. Cancer-specific survival in tissue microarray was assessed using Kaplan-Meier method and compared with the log-rank test.

## SUPPLEMENTARY FIGURES AND TABLE


