# Supplementary material for: Distinct effects of SIRT1 in cancer and stromal cells on tumor promotion
Source: Oncotarget. 2016 Mar 14;7(17):23975–87. doi: 10.18632/oncotarget.8073 (PMC5029678; doi:10.18632/oncotarget.8073)
Supplement: Supplementary file 1 [file oncotarget-07-23975-s001.pdf]

Distinct effects of SIRT1 in cancer and stromal cells on tumor promotion

SUPPLEMENTARY FIGURES AND TABLE

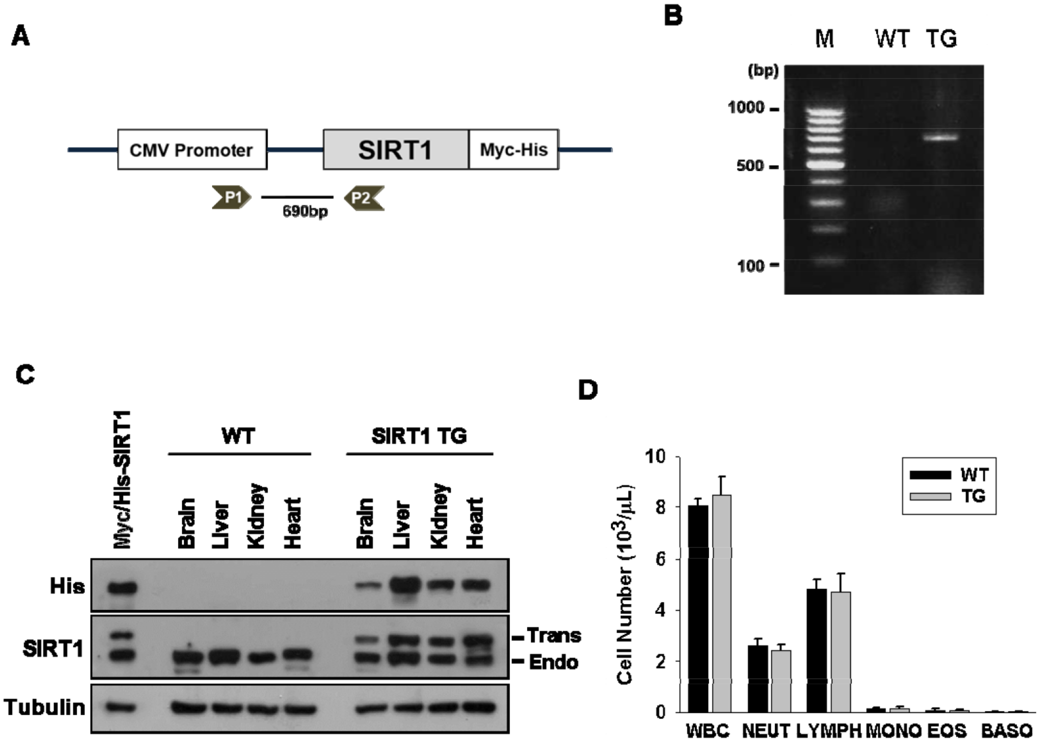

Supplementary Figure S1: A. Schematic representation of the SIRT1 transgene. B. Genotyping of WT and TG mice with the primers indicated in A. The PCR product is 650 bp. **C.** Western blot for checking SIRT1 expression in brains, livers, kidneys, and hearts of WT and TG mice. Endo, endogenous SIRT1; Trans, transgenic SIRT1. **D.** White blood cells (WBC), neutrophils (NEUT), lymphocytes (LYMPH), monocytes (MONO), eosinophils (EOS) and basophils (BASO) were isolated from the blood of WT and TG mice, and were analyzed by flow cytometry (means + s.d.; n=4).

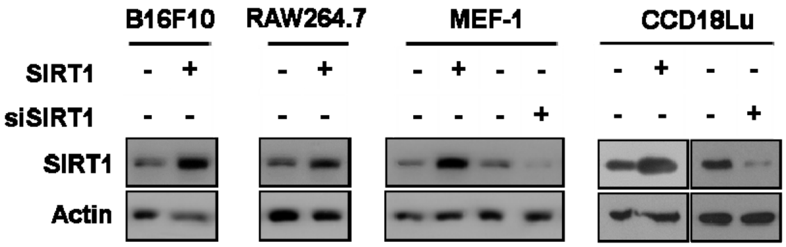

Supplementary Figure S2: B16F10, RAW264.7, MEF-1, and CCD18Lu cells were transfected by SIRT1 (2 µg) and/or siSIRT1 (80 nM), and SIRT1 expression was checked by Western blotting.

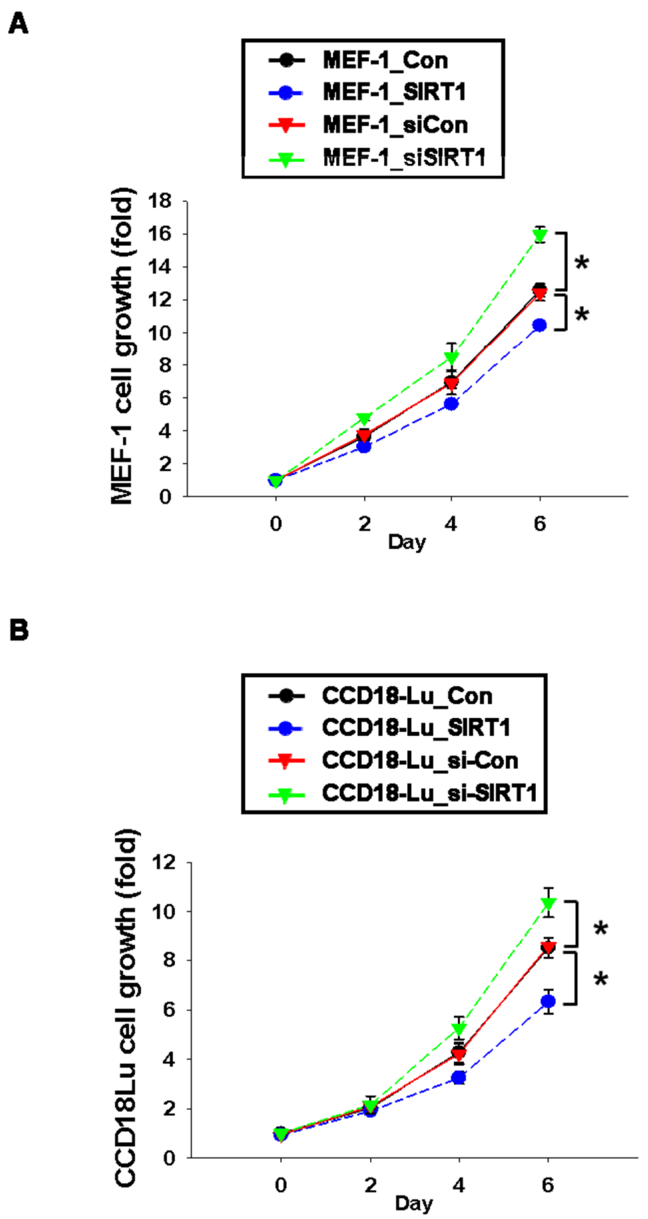

Supplementary Figure S3: Fibroblasts (MEF-1 or CCD19Lu) were transfected by SIRT1 (2 µg) or siSIRT1 (80 nM), and cell growth was checked by MTT analysis every other day for 6 days. Each dot represents the mean ± s.d. (n=4). *, $p < 0.05$.

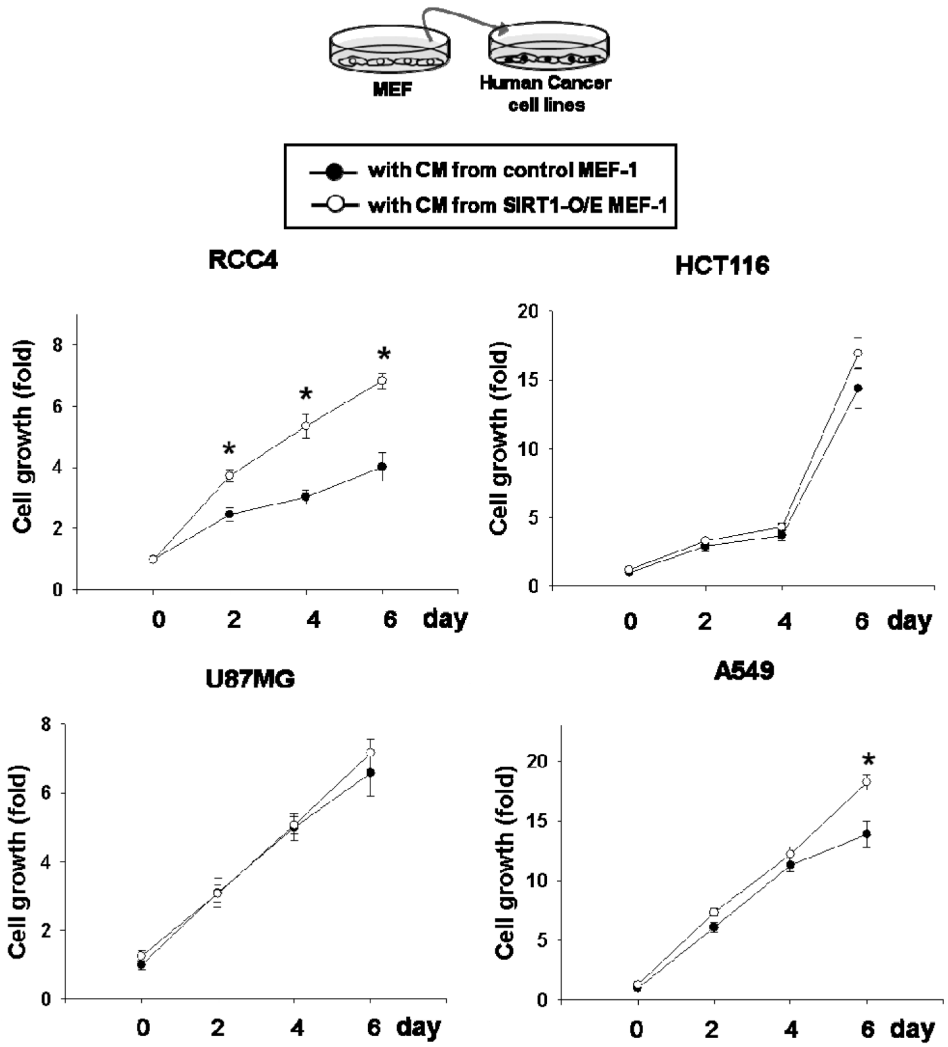

Supplementary Figure S4: The conditioned media were collected from 2 day-cultured MEF-1 and NIH3T3 cells transfected by pcDNA or SIRT1 plasmid. RCC4, HCT116, U87MG, and A549 cells were cultured in the mixture (v:v, 1:1) of the collected media and fresh media. Cell growth was analyzed using MTT every other day for 6 days. Each dot represents the mean ± s.d. from four independent experiments, and * denotes $p < 0.05$.

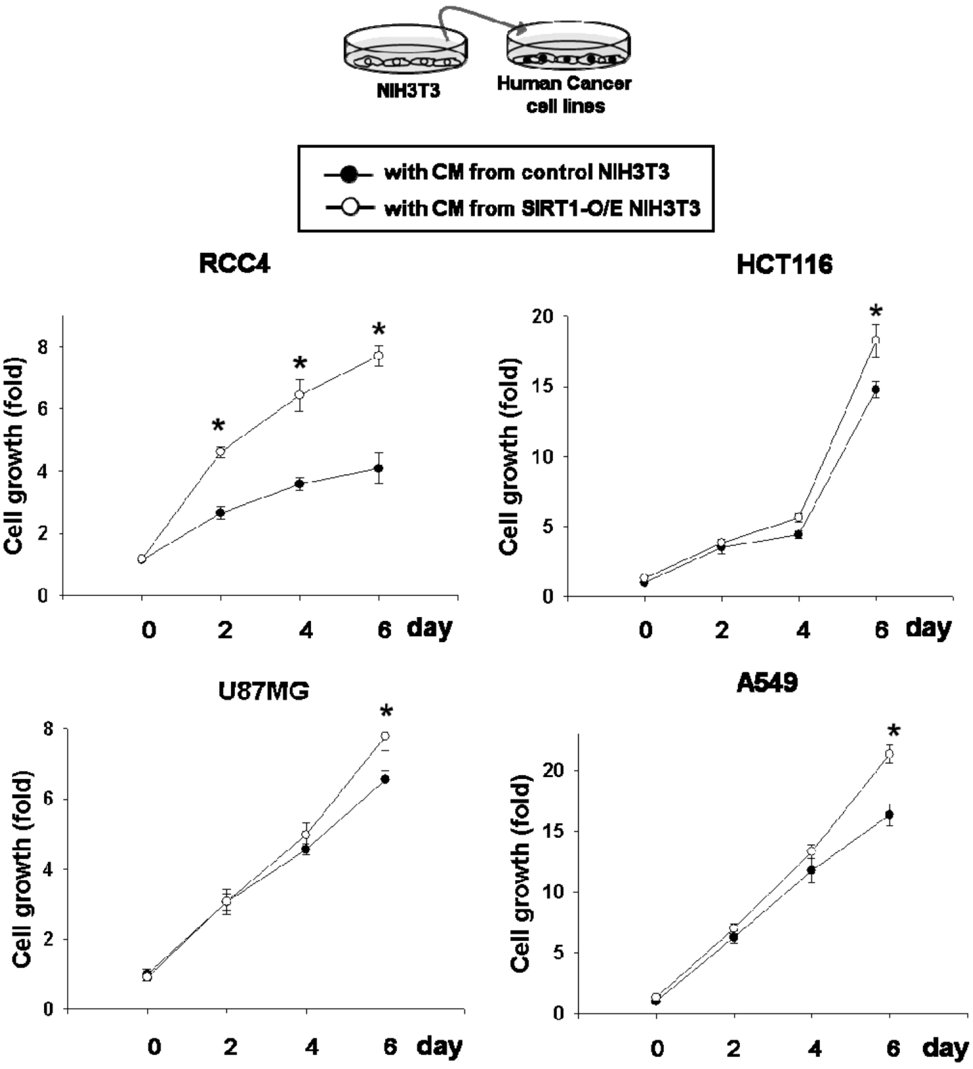

Supplementary Figure S5: The conditioned media were collected from 2 day-cultured MEF-1 and NIH3T3 cells transfected by pcDNA or SIRT1 plasmid. RCC4, HCT116, U87MG, and A549 cells were cultured in the mixture (v:v, 1:1) of the collected media and fresh media. Cell growth was analyzed using MTT every other day for 6 days. Each dot represents the mean ± s.d. from four independent experiments, and * denotes $p < 0.05$.

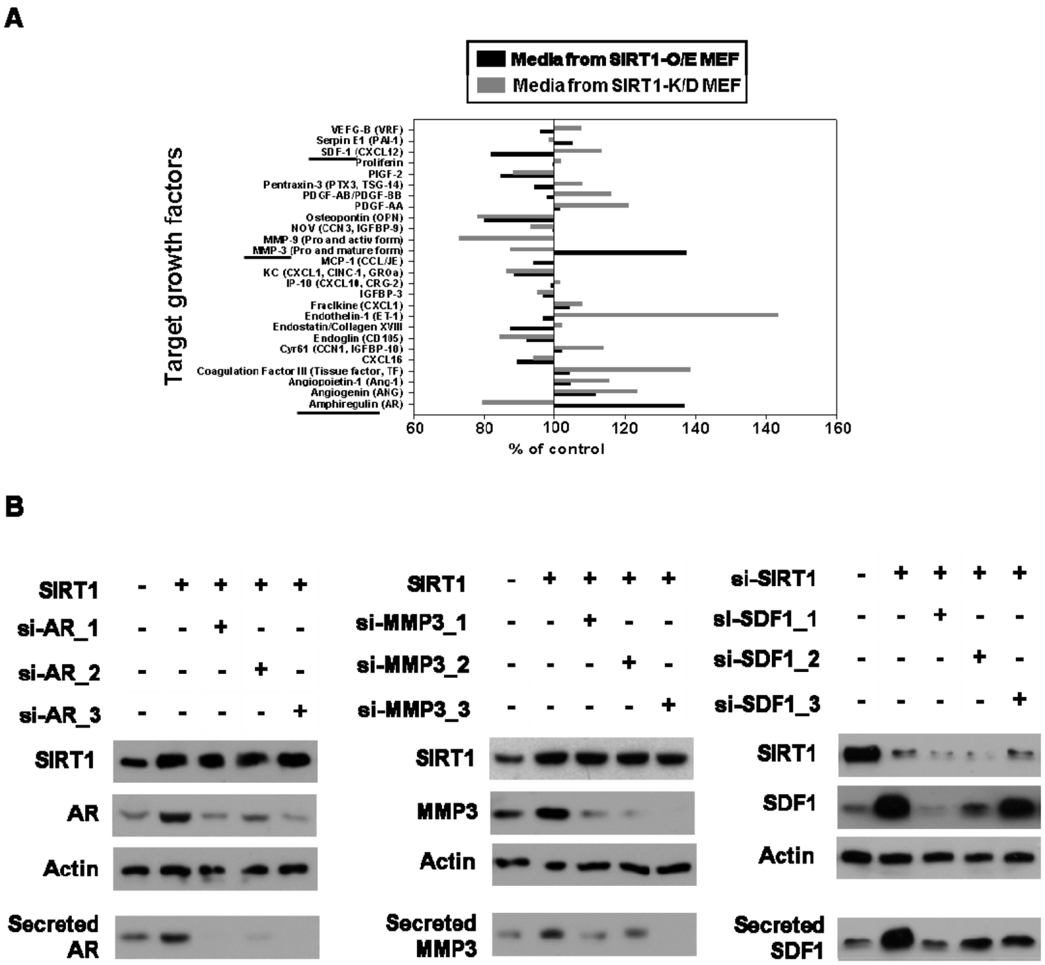

Supplementary Figure S6: A. the average net optical intensity for each pair of spots is presented as percents of the control values. Original film image is shown in Figure 3A. **B.** the expressions of AR, MMP3 or SDF-1 in MEF-1 cells, which had been trasfected with the indicated siRNA, were checked by Western blotting (upper three panels). Secreted AR1, MMP3 or SDF1 was detected in the conditioned media by Western blotting (bottom panel).

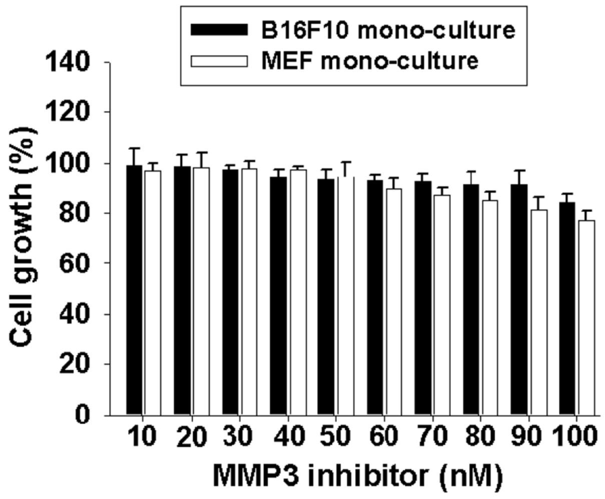

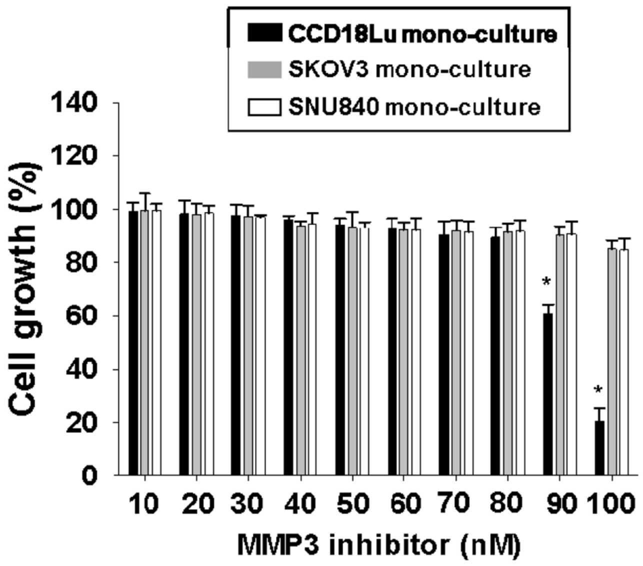

Supplementary Figure S7: Effect of MMP3 inhibitor on cell growth. Murine cells (B16F10 and MEF) or human cells (SKOV3, SNU840, and CCD18Lu) were cultured with MMP3 inhibitor at the indicated concentrations for 6 days, and cell growth was analyzed using MTT staining. * denotes $p < 0.05$ versus the untreated control.

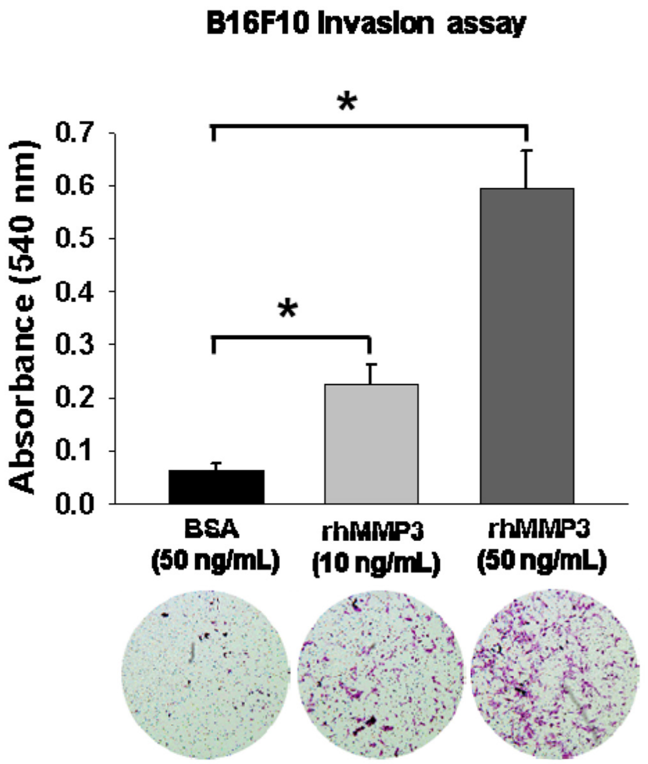

Supplementary Figure S8: The cell invasion assay was performed using a transwell chamber with an 8.0-µm pore membrane coated with matrigel (10 mg/ml, upper surface) and collagen (0.5 mg/ml, lower surface). B16F10 cells (5x10^4) in serum-free media were loaded into the top chamber, and recombinant human MMP3 (rhMMP3) or bovine serum albumin (BSA) was administered as a chemoattractant into the bottom chamber. After incubated at 37°C for 72 h, the cells on the upper surface of the membrane were removed with cotton tips, and the cells on the lower surface of the membrane were stained with 0.1% crystal violet. Crystal violet bound to cells was eluted with acetic acid, and its concentration was quantified at 540 nm. Each experiment was performed in triplicate and results are expressed as the means + s.d. from four independent experiments. *, $p < 0.05$. The bottom panel shows the representative pictures of membrane downside containing stained cells.

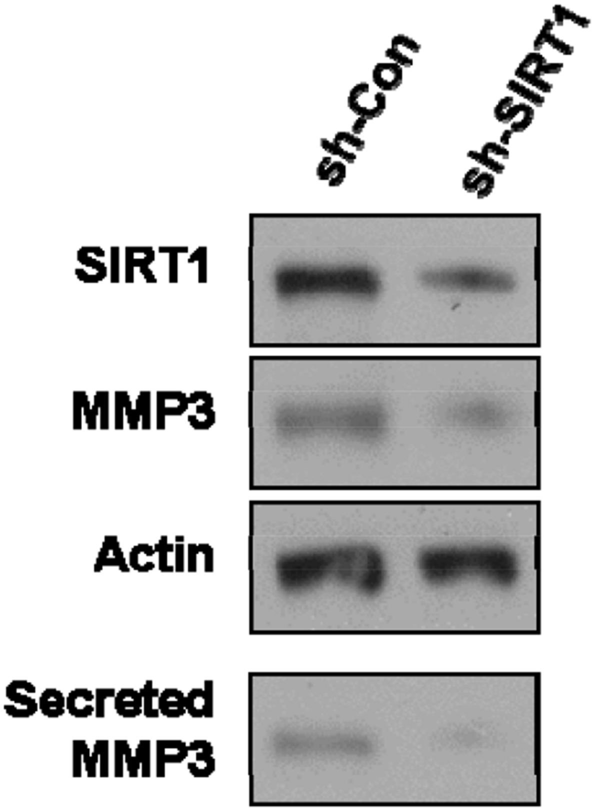

Supplementary Figure S9: MEF cells obtained from WT mice were infected with sh-LTviral-Control or -SIRT1 (0.9 x 108 TU/mL) for 72 hour. The intracellular levels of SIRT1 and MMP3 and the MMP3 level in conditioned medium were analyzed using Western blotting.

Supplementary Table S1: Nucleotide sequences of siRNAs

Purposes	Targets	Sequences (5' to 3')
siRNAs	Non-targeting	UUGAGCAAUUCACGUUCAUUU
	mouse SIRT1	UUCAACAUUCCUACUAGUCUGUACUUC
	mouse MMP3_1	ACCUGAGACAUCACCAAUGUGCAGC
	mouse MMP3_2	GGAAAUCAGUUCUGGGCUAUACGAG
	mouse MMP3_3	AGAGCAAUAGCUGGUUUAAUUGUUA
	mouse AR_1	GCAAUUGUCAUCAAGAUUACUUUGG
	mouse AR_2	GUAUGAUAAUGAACCACAAAUAUCC
	mouse AR_3	CGAAUGCAGAUACAUCGAGAACCUC
	mouse SDF1_1	UGCAUUGACCCGAAAUUAAAGUGGA
	mouse SDF1_2	GCCCUUCAGAUUGUUGCACGGCUGA
	mouse SDF1_3	UACCUGGAGAAAGCUUUAAACAAGU
	human SIRT1	UUCAACAUUCCUAGAAGUUUGUACUUC
	human MMP3	GAGUUUGACCCAAAUGCAAAGAAAGAG
